# Poly[(μ-4,4′-bipyridine)(μ-naphthalene-1,4-dicarboxyl­ato)manganese(II)]

**DOI:** 10.1107/S1600536809009301

**Published:** 2009-03-25

**Authors:** Jan Boeckmann, Inke Jess, Christian Näther

**Affiliations:** a Institut für Anorganische Chemie, Christian-Albrechts-Universität Kiel, Max-Eyth Strasse 2, D-24098 Kiel, Germany

## Abstract

In the crystal structure of the title compound, [Mn(C_12_H_6_O_4_)(C_10_H_8_N_2_)]_*n*_, the Mn atoms are each coordinated by four O atoms of naphthalene-1,4-dicarboxyl­ate anions and two N atoms of two symmetry-related 4,4′-bipyridine ligands within a strongly distorted octa­hedra. Two of the O atoms originate from one naphthalene-1,4-dicarboxyl­ate anion, whereas the remaining two O atoms derive from two symmetry-equivalent naphthalene-1,4-dicarboxyl­ate anions. Two Mn atoms are connected *via* the anions into dimers, which are further linked by the anions and the N-donor ligands into a three-dimensional coordination network.

## Related literature

For the isotypic structure with Fe^II^, see Boeckmann *et al.* (2009 ▶). For related structures, see Zheng *et al.* (2005 ▶).
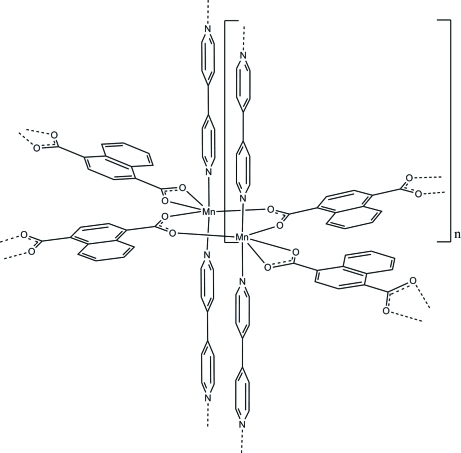


## Experimental

### 

#### Crystal data

[Mn(C_12_H_6_O_4_)(C_10_H_8_N_2_)]
*M* *_r_* = 425.29
Monoclinic, 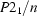

*a* = 10.5567 (3) Å
*b* = 30.1870 (6) Å
*c* = 11.6879 (3) Å
β = 93.734 (2)°
*V* = 3716.74 (16) Å^3^
*Z* = 8
Mo *K*α radiation
μ = 0.74 mm^−1^
*T* = 293 K
0.14 × 0.08 × 0.06 mm

#### Data collection

Stoe IPDS-II diffractometer
Absorption correction: numerical (*X-SHAPE* and *X-RED32*; Stoe & Cie, 2008 ▶) *T*_min_ = 0.929, *T*_max_ = 0.953
45141 measured reflections
7902 independent reflections
6545 reflections with *I* > 2σ(*I*)
*R*_int_ = 0.057

#### Refinement

*R*[*F* ^2^ > 2σ(*F* ^2^)] = 0.063
*wR*(*F* ^2^) = 0.119
*S* = 1.17
7902 reflections
523 parameters
H-atom parameters constrained
Δρ_max_ = 0.31 e Å^−3^
Δρ_min_ = −0.36 e Å^−3^

### 

Data collection: *X-AREA* (Stoe & Cie, 2008 ▶); cell refinement: *X-AREA*; data reduction: *X-AREA*; program(s) used to solve structure: *SHELXS97* (Sheldrick, 2008 ▶); program(s) used to refine structure: *SHELXL97* (Sheldrick, 2008 ▶); molecular graphics: *DIAMOND* (Brandenburg, 2008 ▶) and *XP* in *SHELXTL* (Sheldrick, 2008 ▶); software used to prepare material for publication: *XCIF* in *SHELXTL*.

## Supplementary Material

Crystal structure: contains datablocks I, global. DOI: 10.1107/S1600536809009301/bt2868sup1.cif

Structure factors: contains datablocks I. DOI: 10.1107/S1600536809009301/bt2868Isup2.hkl

Additional supplementary materials:  crystallographic information; 3D view; checkCIF report

## Figures and Tables

**Fig. 1.**
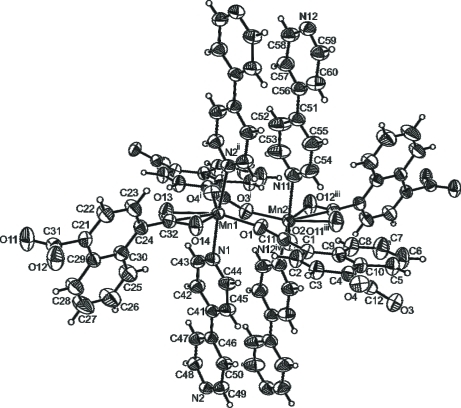
: Crystal structure of the title compound with labelling and displacement ellipsoids drawn at the 50% probability level. Symmetry codes: i = x + 1, y, z; ii = x, y, z + 1; iii = -x + 1.5, y - 1/2, -z + 1.5; iv = x, y, z - 1.

**Fig. 2.**
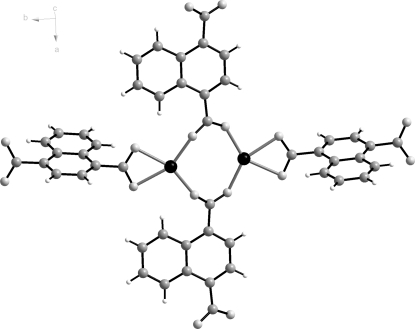
: Crystal structure of the title compound with view onto the dimers. The 4,4'-bipyridine molecules are omitted for clarity.

**Fig. 3.**
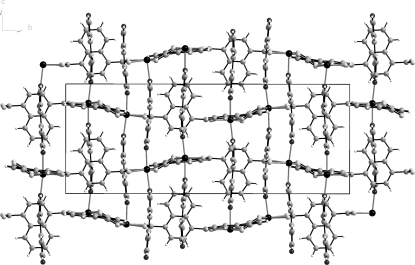
: Crystal structure of the title compound with view in the direction of the crystallographic a axis.

## supplementary crystallographic information

### Comment

In our ongoing investigations on the synthesis of new metal-organic frameworks, we prepared the title compound, C_22_H_14_N_2_O_4_Mn, by the reaction of manganese(II)chloride with naphthalene-1,4-dicarboxylic acid in sodium hydroxide, sodium acetate, 4,4'- bipyridine and water. Poly[(µ-4,4'-bipyridine)(µ-naphthalene-1,4-dicarboxylato) manganese(II)] is isotypic to Poly[(µ-4,4'-bipyridine)(µ-naphthalene-1,4-dicarboxylato) iron(II)], reported recently (Boeckmann *et al.*,  2009).

In the crystal structure of the title compound the manganese atoms are each surrounded by four O atoms of three symmetry related naphthalene-1,4-dicarboxylate anions and two N atoms of two 4,4'-bipyridine ligands related by symmetry within a distorted octahedral coordination environment (Fig 1). Two symmetry equivalent naphthalene-1,4-dicarboxylate anions bridges two symmetry related manganese atoms into dimers, which is located on a centre of inversion (Fig 2). Such dimers are also found in the structure of [Eu_2_(NDC)_3_(4,4'-bipyridine)_0.5_(H_2_O)_3_] ^.^ (4,4'-bipyridine) (Zheng *et al.*, 2005). These dimanganese(II)-centered octahedra are connected *via* the naphthalene-1,4-dicarboxylate anions into layers, which are parallel to the a/b plane, and are further connected by the 4,4'-bipyridine ligands into a three-dimensional coordination network (Fig 3).

### Experimental

16.2 mg MnCl_2_^.^ 2 H_2_O (0.10 mmol), 33.0 mg naphthalene-1,4-dicarboxylic acid (0.15 mmol), 10.4 mg NaOH (0.26 mmol), 40.0 mg NaAc ^.^ 3 H_2_O (0.30 mmol), 20.0 mg 4,4'-Bipyridine (0.10 mmol) and 5 ml of water were transfered into a glass tube and heated to 150° C for 4 d. On cooling colourless blocks of the title compound were obtained.

### Refinement

All H atoms were located in difference map but were positioned with idealized geometry and were refined isotropic with *U*_eq_(H) = 1.2 *U*_eq_(C) of the parent atom using a riding model with C—H = 0.93 Å.

### Crystal data

**Table:**

[Mn(C_12_H_6_O_4_)(C_10_H_8_N_2_)]	*F*(000) = 1736
*M**_r_* = 425.29	*D*_x_ = 1.520 Mg m^−^^3^
Monoclinic, *P*2_1_/*n*	Mo *K*α radiation, λ = 0.71073 Å
*a* = 10.5567 (3) Å	Cell parameters from 43887 reflections
*b* = 30.1870 (6) Å	θ = 1.4–27.2°
*c* = 11.6879 (3) Å	µ = 0.74 mm^−^^1^
β = 93.734 (2)°	*T* = 293 K
*V* = 3716.74 (16) Å^3^	Blocks, colourless
*Z* = 8	0.14 × 0.08 × 0.06 mm

### Data collection

**Table:**

Stoe IPDS-II diffractometer	7902 independent reflections
Radiation source: fine-focus sealed tube	6545 reflections with *I* > 2σ(*I*)
graphite	*R*_int_ = 0.057
Detector resolution: 0.150 pixels mm^-1^	θ_max_ = 26.8°, θ_min_ = 1.4°
ω scans	*h* = −12→13
Absorption correction: numerical (*X-SHAPE* and *X-RED32*; Stoe & Cie, 2008)	*k* = −38→38
*T*_min_ = 0.929, *T*_max_ = 0.953	*l* = −14→14
45141 measured reflections	

### Refinement

**Table:**

Refinement on *F*^2^	Primary atom site location: structure-invariant direct methods
Least-squares matrix: full	Secondary atom site location: difference Fourier map
*R*[*F*^2^ > 2σ(*F*^2^)] = 0.063	Hydrogen site location: inferred from neighbouring sites
*wR*(*F*^2^) = 0.119	H-atom parameters constrained
*S* = 1.17	*w* = 1/[σ^2^(*F*_o_^2^) + (0.0296*P*)^2^ + 4.295*P*] where *P* = (*F*_o_^2^ + 2*F*_c_^2^)/3
7902 reflections	(Δ/σ)_max_ = 0.001
523 parameters	Δρ_max_ = 0.31 e Å^−^^3^
0 restraints	Δρ_min_ = −0.35 e Å^−^^3^

### Special details

**Table:**

Geometry. All e.s.d.'s (except the e.s.d. in the dihedral angle between two l.s. planes) are estimated using the full covariance matrix. The cell e.s.d.'s are taken into account individually in the estimation of e.s.d.'s in distances, angles and torsion angles; correlations between e.s.d.'s in cell parameters are only used when they are defined by crystal symmetry. An approximate (isotropic) treatment of cell e.s.d.'s is used for estimating e.s.d.'s involving l.s. planes.
Refinement. Refinement of *F*^2^ against ALL reflections. The weighted *R*-factor *wR* and goodness of fit *S* are based on *F*^2^, conventional *R*-factors *R* are based on *F*, with *F* set to zero for negative *F*^2^. The threshold expression of *F*^2^ > σ(*F*^2^) is used only for calculating *R*-factors(gt) *etc*. and is not relevant to the choice of reflections for refinement. *R*-factors based on *F*^2^ are statistically about twice as large as those based on *F*, and *R*- factors based on ALL data will be even larger.

### Fractional atomic coordinates and isotropic or equivalent isotropic displacement parameters (Å^2^)

**Table:**

	*x*	*y*	*z*	*U*_iso_*/*U*_eq_	
Mn1	0.77819 (5)	0.714006 (14)	0.77976 (4)	0.03123 (12)	
Mn2	0.77185 (5)	0.579806 (15)	0.67513 (4)	0.03472 (13)	
C1	0.4084 (3)	0.64521 (10)	0.7166 (3)	0.0379 (7)	
C2	0.3428 (3)	0.68342 (11)	0.7357 (3)	0.0461 (8)	
H2	0.3871	0.7099	0.7451	0.055*	
C3	0.2103 (3)	0.68348 (11)	0.7413 (3)	0.0461 (8)	
H3	0.1691	0.7102	0.7529	0.055*	
C4	0.1404 (3)	0.64549 (10)	0.7302 (3)	0.0373 (7)	
C5	0.1409 (4)	0.56271 (11)	0.7086 (3)	0.0495 (9)	
H5	0.0536	0.5621	0.7151	0.059*	
C6	0.2032 (4)	0.52440 (12)	0.6950 (4)	0.0642 (12)	
H6	0.1587	0.4978	0.6918	0.077*	
C7	0.3352 (4)	0.52436 (13)	0.6856 (4)	0.0680 (12)	
H7	0.3778	0.4977	0.6762	0.082*	
C8	0.4007 (4)	0.56295 (11)	0.6901 (4)	0.0523 (9)	
H8	0.4880	0.5624	0.6832	0.063*	
C9	0.3398 (3)	0.60406 (10)	0.7049 (3)	0.0387 (7)	
C10	0.2049 (3)	0.60412 (10)	0.7135 (3)	0.0386 (7)	
C11	0.5509 (3)	0.64903 (10)	0.7160 (3)	0.0352 (7)	
O1	0.6003 (2)	0.68148 (8)	0.7687 (2)	0.0457 (6)	
O2	0.6110 (2)	0.62049 (8)	0.6641 (2)	0.0463 (6)	
C12	−0.0020 (3)	0.64949 (10)	0.7354 (3)	0.0368 (7)	
O3	−0.0702 (2)	0.62131 (8)	0.6830 (2)	0.0501 (6)	
O4	−0.0424 (2)	0.68245 (8)	0.7874 (2)	0.0458 (6)	
C21	0.7452 (3)	0.94371 (10)	0.8155 (3)	0.0389 (7)	
C22	0.7282 (4)	0.91782 (11)	0.9088 (3)	0.0498 (9)	
H22	0.7105	0.9311	0.9778	0.060*	
C23	0.7368 (4)	0.87126 (11)	0.9028 (3)	0.0509 (9)	
H23	0.7239	0.8544	0.9676	0.061*	
C24	0.7635 (3)	0.85071 (10)	0.8037 (3)	0.0386 (7)	
C25	0.8072 (5)	0.85703 (13)	0.5973 (3)	0.0615 (11)	
H25	0.8148	0.8264	0.5921	0.074*	
C26	0.8203 (6)	0.88213 (15)	0.5031 (4)	0.0802 (16)	
H26	0.8366	0.8686	0.4342	0.096*	
C27	0.8095 (6)	0.92831 (15)	0.5079 (4)	0.0797 (15)	
H27	0.8181	0.9452	0.4423	0.096*	
C28	0.7865 (4)	0.94860 (13)	0.6088 (3)	0.0599 (11)	
H28	0.7802	0.9793	0.6114	0.072*	
C29	0.7719 (3)	0.92355 (10)	0.7098 (3)	0.0400 (7)	
C30	0.7822 (3)	0.87639 (11)	0.7040 (3)	0.0412 (7)	
C31	0.7350 (3)	0.99366 (11)	0.8236 (3)	0.0408 (7)	
O11	0.8342 (3)	1.01602 (8)	0.8250 (3)	0.0649 (8)	
O12	0.6298 (3)	1.01132 (8)	0.8259 (3)	0.0669 (8)	
C32	0.7718 (3)	0.80079 (10)	0.7979 (3)	0.0373 (7)	
O13	0.8766 (2)	0.78240 (8)	0.7851 (2)	0.0543 (7)	
O14	0.6728 (2)	0.77859 (7)	0.8027 (2)	0.0507 (6)	
C41	0.7782 (3)	0.70738 (10)	0.3422 (3)	0.0364 (7)	
C42	0.8900 (3)	0.71117 (12)	0.4116 (3)	0.0460 (8)	
H42	0.9681	0.7108	0.3790	0.055*	
C43	0.8846 (3)	0.71547 (12)	0.5288 (3)	0.0456 (8)	
H43	0.9608	0.7179	0.5729	0.055*	
N1	0.7771 (3)	0.71640 (9)	0.5828 (2)	0.0416 (6)	
C44	0.6697 (3)	0.71320 (12)	0.5159 (3)	0.0446 (8)	
H44	0.5931	0.7140	0.5509	0.054*	
C45	0.6657 (3)	0.70878 (12)	0.3975 (3)	0.0457 (8)	
H45	0.5881	0.7068	0.3554	0.055*	
C46	0.7791 (3)	0.70395 (10)	0.2152 (3)	0.0352 (7)	
C47	0.8908 (3)	0.70268 (12)	0.1596 (3)	0.0448 (8)	
H47	0.9685	0.7019	0.2019	0.054*	
C48	0.8870 (3)	0.70257 (12)	0.0409 (3)	0.0441 (8)	
H48	0.9636	0.7015	0.0058	0.053*	
N2	0.7797 (3)	0.70391 (9)	−0.0260 (2)	0.0381 (6)	
C49	0.6711 (3)	0.70379 (12)	0.0276 (3)	0.0451 (8)	
H49	0.5948	0.7037	−0.0170	0.054*	
C50	0.6665 (3)	0.70380 (12)	0.1455 (3)	0.0437 (8)	
H50	0.5886	0.7037	0.1784	0.052*	
C51	0.7779 (4)	0.58616 (12)	1.1149 (3)	0.0479 (9)	
C52	0.8887 (5)	0.58533 (18)	1.0592 (3)	0.0730 (13)	
H52	0.9663	0.5869	1.1014	0.088*	
C53	0.8857 (5)	0.58219 (18)	0.9417 (3)	0.0723 (13)	
H53	0.9626	0.5818	0.9071	0.087*	
N11	0.7789 (4)	0.57972 (10)	0.8742 (3)	0.0545 (8)	
C54	0.6726 (5)	0.58022 (16)	0.9282 (3)	0.0672 (12)	
H54	0.5961	0.5784	0.8841	0.081*	
C55	0.6677 (4)	0.58323 (17)	1.0459 (3)	0.0678 (12)	
H55	0.5897	0.5833	1.0785	0.081*	
C56	0.7762 (4)	0.58964 (11)	1.2420 (3)	0.0449 (8)	
C57	0.8866 (4)	0.58875 (13)	1.3122 (3)	0.0534 (9)	
H57	0.9650	0.5885	1.2802	0.064*	
C58	0.8804 (4)	0.58820 (13)	1.4301 (3)	0.0530 (9)	
H58	0.9561	0.5878	1.4755	0.064*	
N12	0.7719 (3)	0.58829 (10)	1.4818 (2)	0.0443 (7)	
C59	0.6656 (4)	0.59161 (13)	1.4149 (3)	0.0511 (9)	
H59	0.5887	0.5934	1.4494	0.061*	
C60	0.6638 (4)	0.59256 (13)	1.2964 (3)	0.0526 (9)	
H60	0.5870	0.5952	1.2532	0.063*	

### Atomic displacement parameters (Å^2^)

**Table:**

	*U*^11^	*U*^22^	*U*^33^	*U*^12^	*U*^13^	*U*^23^
Mn1	0.0340 (2)	0.0311 (2)	0.0289 (2)	−0.00001 (19)	0.00408 (18)	−0.00152 (18)
Mn2	0.0400 (3)	0.0316 (2)	0.0329 (2)	−0.0005 (2)	0.0045 (2)	−0.00109 (19)
C1	0.0354 (17)	0.0374 (16)	0.0410 (17)	0.0000 (13)	0.0039 (14)	−0.0032 (13)
C2	0.042 (2)	0.0347 (16)	0.062 (2)	−0.0048 (14)	0.0056 (17)	−0.0040 (15)
C3	0.042 (2)	0.0347 (16)	0.063 (2)	0.0022 (14)	0.0083 (17)	−0.0052 (15)
C4	0.0351 (17)	0.0382 (16)	0.0393 (17)	−0.0016 (13)	0.0057 (13)	−0.0017 (13)
C5	0.044 (2)	0.0398 (18)	0.065 (2)	−0.0057 (15)	0.0051 (18)	−0.0009 (16)
C6	0.059 (3)	0.0344 (18)	0.100 (3)	−0.0073 (18)	0.007 (2)	−0.002 (2)
C7	0.060 (3)	0.0341 (19)	0.110 (4)	0.0034 (18)	0.006 (3)	−0.008 (2)
C8	0.042 (2)	0.0408 (18)	0.075 (3)	0.0025 (16)	0.0076 (18)	−0.0083 (17)
C9	0.0406 (19)	0.0347 (16)	0.0414 (18)	0.0005 (14)	0.0060 (14)	−0.0024 (13)
C10	0.0394 (18)	0.0355 (16)	0.0412 (18)	−0.0019 (14)	0.0054 (14)	−0.0016 (13)
C11	0.0350 (17)	0.0364 (16)	0.0348 (16)	−0.0006 (13)	0.0054 (13)	0.0010 (13)
O1	0.0406 (13)	0.0516 (14)	0.0451 (13)	−0.0129 (11)	0.0043 (11)	−0.0106 (11)
O2	0.0399 (14)	0.0461 (13)	0.0538 (14)	0.0064 (11)	0.0099 (11)	−0.0040 (11)
C12	0.0388 (18)	0.0379 (16)	0.0340 (16)	0.0026 (14)	0.0049 (13)	0.0019 (13)
O3	0.0409 (14)	0.0519 (14)	0.0574 (15)	−0.0083 (11)	0.0015 (12)	−0.0073 (12)
O4	0.0443 (14)	0.0509 (14)	0.0427 (13)	0.0124 (11)	0.0061 (11)	−0.0067 (11)
C21	0.0413 (19)	0.0321 (15)	0.0434 (18)	−0.0021 (14)	0.0050 (14)	−0.0012 (13)
C22	0.074 (3)	0.0365 (17)	0.0411 (19)	0.0032 (17)	0.0163 (18)	−0.0036 (14)
C23	0.075 (3)	0.0375 (17)	0.0416 (19)	0.0008 (17)	0.0165 (18)	0.0036 (14)
C24	0.0435 (19)	0.0310 (15)	0.0414 (18)	0.0011 (14)	0.0046 (14)	0.0011 (13)
C25	0.101 (4)	0.044 (2)	0.040 (2)	0.005 (2)	0.013 (2)	−0.0071 (16)
C26	0.144 (5)	0.061 (3)	0.038 (2)	0.006 (3)	0.019 (3)	−0.0062 (19)
C27	0.137 (5)	0.063 (3)	0.040 (2)	0.007 (3)	0.018 (3)	0.0100 (19)
C28	0.094 (3)	0.0423 (19)	0.044 (2)	0.002 (2)	0.010 (2)	0.0055 (16)
C29	0.051 (2)	0.0323 (15)	0.0366 (17)	−0.0012 (14)	0.0050 (15)	0.0009 (13)
C30	0.048 (2)	0.0360 (16)	0.0398 (18)	0.0020 (15)	0.0045 (15)	−0.0024 (13)
C31	0.0459 (19)	0.0382 (16)	0.0388 (17)	0.0023 (15)	0.0062 (14)	0.0027 (14)
O11	0.0525 (17)	0.0349 (13)	0.108 (2)	−0.0048 (12)	0.0090 (16)	−0.0026 (14)
O12	0.0499 (17)	0.0398 (14)	0.111 (2)	0.0052 (12)	0.0075 (16)	−0.0027 (15)
C32	0.0399 (18)	0.0345 (15)	0.0372 (17)	−0.0013 (14)	0.0006 (14)	−0.0009 (13)
O13	0.0397 (14)	0.0378 (13)	0.086 (2)	0.0007 (11)	0.0052 (13)	−0.0063 (12)
O14	0.0414 (14)	0.0322 (12)	0.0793 (18)	−0.0012 (10)	0.0098 (13)	−0.0008 (11)
C41	0.0432 (18)	0.0357 (15)	0.0305 (16)	0.0003 (14)	0.0053 (13)	0.0009 (12)
C42	0.0433 (19)	0.061 (2)	0.0342 (17)	−0.0041 (17)	0.0063 (15)	0.0005 (15)
C43	0.0424 (19)	0.061 (2)	0.0333 (17)	−0.0095 (17)	0.0012 (14)	0.0020 (15)
N1	0.0472 (17)	0.0455 (15)	0.0323 (14)	−0.0010 (13)	0.0037 (12)	0.0007 (12)
C44	0.044 (2)	0.057 (2)	0.0329 (17)	0.0068 (17)	0.0064 (14)	0.0026 (15)
C45	0.0421 (19)	0.062 (2)	0.0330 (17)	0.0031 (17)	0.0018 (14)	0.0022 (15)
C46	0.0399 (17)	0.0362 (16)	0.0297 (15)	0.0021 (13)	0.0036 (13)	−0.0003 (12)
C47	0.0386 (19)	0.059 (2)	0.0371 (18)	0.0022 (16)	0.0025 (14)	−0.0031 (15)
C48	0.0404 (19)	0.056 (2)	0.0363 (17)	−0.0008 (16)	0.0069 (15)	−0.0033 (15)
N2	0.0432 (16)	0.0412 (14)	0.0302 (13)	0.0019 (12)	0.0040 (12)	0.0005 (11)
C49	0.0395 (19)	0.060 (2)	0.0354 (17)	0.0020 (16)	0.0018 (14)	−0.0024 (15)
C50	0.0423 (19)	0.056 (2)	0.0333 (17)	0.0012 (16)	0.0057 (14)	−0.0010 (15)
C51	0.067 (2)	0.0435 (18)	0.0336 (18)	0.0036 (17)	0.0047 (17)	−0.0003 (14)
C52	0.066 (3)	0.119 (4)	0.034 (2)	0.013 (3)	0.0024 (19)	−0.003 (2)
C53	0.071 (3)	0.110 (4)	0.038 (2)	0.018 (3)	0.009 (2)	0.002 (2)
N11	0.078 (2)	0.0488 (17)	0.0371 (16)	0.0022 (17)	0.0070 (16)	−0.0021 (13)
C54	0.073 (3)	0.093 (3)	0.035 (2)	−0.017 (3)	−0.0013 (19)	−0.002 (2)
C55	0.063 (3)	0.105 (4)	0.035 (2)	−0.010 (3)	0.0047 (19)	−0.003 (2)
C56	0.059 (2)	0.0411 (17)	0.0342 (17)	0.0021 (16)	0.0013 (16)	−0.0009 (14)
C57	0.058 (2)	0.065 (2)	0.0385 (19)	−0.0017 (19)	0.0080 (17)	−0.0020 (17)
C58	0.055 (2)	0.068 (2)	0.0354 (18)	0.0002 (19)	0.0027 (16)	−0.0038 (17)
N12	0.0509 (18)	0.0480 (16)	0.0342 (15)	0.0017 (14)	0.0048 (13)	−0.0009 (12)
C59	0.054 (2)	0.062 (2)	0.0375 (19)	0.0018 (19)	0.0056 (17)	−0.0033 (16)
C60	0.059 (2)	0.062 (2)	0.0368 (19)	0.0065 (19)	0.0006 (17)	−0.0018 (16)

### Geometric parameters (Å, °)

**Table:**

Mn1—O1	2.115 (2)	C28—C29	1.418 (5)
Mn1—O4^i^	2.116 (2)	C28—H28	0.9300
Mn1—O14	2.269 (2)	C29—C30	1.430 (4)
Mn1—N2^ii^	2.290 (3)	C31—O12	1.234 (4)
Mn1—N1	2.302 (3)	C31—O11	1.245 (4)
Mn1—O13	2.310 (2)	C31—Mn2^vi^	2.602 (3)
Mn1—C32	2.629 (3)	O11—Mn2^vi^	2.227 (3)
Mn2—O3^i^	2.083 (2)	O12—Mn2^vi^	2.314 (3)
Mn2—O2	2.093 (2)	C32—O14	1.246 (4)
Mn2—O11^iii^	2.227 (3)	C32—O13	1.255 (4)
Mn2—N12^iv^	2.274 (3)	C41—C45	1.389 (5)
Mn2—O12^iii^	2.314 (3)	C41—C42	1.392 (5)
Mn2—N11	2.323 (3)	C41—C46	1.489 (4)
Mn2—C31^iii^	2.602 (3)	C42—C43	1.381 (5)
C1—C2	1.371 (4)	C42—H42	0.9300
C1—C9	1.440 (4)	C43—N1	1.334 (4)
C1—C11	1.510 (4)	C43—H43	0.9300
C2—C3	1.404 (5)	N1—C44	1.338 (4)
C2—H2	0.9300	C44—C45	1.389 (4)
C3—C4	1.365 (4)	C44—H44	0.9300
C3—H3	0.9300	C45—H45	0.9300
C4—C10	1.442 (4)	C46—C47	1.384 (4)
C4—C12	1.514 (4)	C46—C50	1.397 (5)
C5—C6	1.345 (5)	C47—C48	1.386 (5)
C5—C10	1.420 (5)	C47—H47	0.9300
C5—H5	0.9300	C48—N2	1.334 (4)
C6—C7	1.406 (6)	C48—H48	0.9300
C6—H6	0.9300	N2—C49	1.341 (4)
C7—C8	1.354 (5)	N2—Mn1^iv^	2.290 (3)
C7—H7	0.9300	C49—C50	1.383 (4)
C8—C9	1.413 (4)	C49—H49	0.9300
C8—H8	0.9300	C50—H50	0.9300
C9—C10	1.435 (5)	C51—C55	1.374 (6)
C11—O2	1.251 (4)	C51—C52	1.375 (6)
C11—O1	1.253 (4)	C51—C56	1.490 (5)
C12—O3	1.248 (4)	C52—C53	1.375 (5)
C12—O4	1.255 (4)	C52—H52	0.9300
O3—Mn2^v^	2.083 (2)	C53—N11	1.335 (6)
O4—Mn1^v^	2.116 (2)	C53—H53	0.9300
C21—C22	1.364 (5)	N11—C54	1.323 (5)
C21—C29	1.421 (4)	C54—C55	1.383 (5)
C21—C31	1.515 (4)	C54—H54	0.9300
C22—C23	1.411 (5)	C55—H55	0.9300
C22—H22	0.9300	C56—C57	1.381 (5)
C23—C24	1.360 (5)	C56—C60	1.385 (5)
C23—H23	0.9300	C57—C58	1.384 (5)
C24—C30	1.424 (4)	C57—H57	0.9300
C24—C32	1.511 (4)	C58—N12	1.330 (5)
C25—C26	1.351 (6)	C58—H58	0.9300
C25—C30	1.417 (5)	N12—C59	1.329 (5)
C25—H25	0.9300	N12—Mn2^ii^	2.274 (3)
C26—C27	1.400 (6)	C59—C60	1.385 (5)
C26—H26	0.9300	C59—H59	0.9300
C27—C28	1.365 (6)	C60—H60	0.9300
C27—H27	0.9300		
			
O1—Mn1—O4^i^	125.59 (10)	C28—C27—C26	120.1 (4)
O1—Mn1—O14	88.05 (9)	C28—C27—H27	120.0
O4^i^—Mn1—O14	145.67 (10)	C26—C27—H27	120.0
O1—Mn1—N2^ii^	86.99 (10)	C27—C28—C29	121.0 (4)
O4^i^—Mn1—N2^ii^	87.11 (9)	C27—C28—H28	119.5
O14—Mn1—N2^ii^	88.22 (10)	C29—C28—H28	119.5
O1—Mn1—N1	90.41 (10)	C28—C29—C21	122.2 (3)
O4^i^—Mn1—N1	90.15 (10)	C28—C29—C30	118.6 (3)
O14—Mn1—N1	96.94 (10)	C21—C29—C30	119.2 (3)
N2^ii^—Mn1—N1	174.15 (10)	C25—C30—C24	122.6 (3)
O1—Mn1—O13	144.32 (9)	C25—C30—C29	118.1 (3)
O4^i^—Mn1—O13	90.08 (9)	C24—C30—C29	119.2 (3)
O14—Mn1—O13	56.77 (9)	O12—C31—O11	121.5 (3)
N2^ii^—Mn1—O13	96.79 (10)	O12—C31—C21	119.9 (3)
N1—Mn1—O13	88.38 (10)	O11—C31—C21	118.5 (3)
O1—Mn1—C32	116.11 (10)	O12—C31—Mn2^vi^	62.78 (18)
O4^i^—Mn1—C32	118.19 (10)	O11—C31—Mn2^vi^	58.79 (18)
O14—Mn1—C32	28.26 (9)	C21—C31—Mn2^vi^	175.7 (2)
N2^ii^—Mn1—C32	92.94 (10)	C31—O11—Mn2^vi^	92.7 (2)
N1—Mn1—C32	92.91 (10)	C31—O12—Mn2^vi^	88.9 (2)
O13—Mn1—C32	28.52 (9)	O14—C32—O13	121.1 (3)
O3^i^—Mn2—O2	107.09 (10)	O14—C32—C24	118.9 (3)
O3^i^—Mn2—O11^iii^	157.06 (10)	O13—C32—C24	120.0 (3)
O2—Mn2—O11^iii^	95.82 (10)	O14—C32—Mn1	59.59 (16)
O3^i^—Mn2—N12^iv^	85.66 (11)	O13—C32—Mn1	61.49 (17)
O2—Mn2—N12^iv^	85.71 (10)	C24—C32—Mn1	177.3 (2)
O11^iii^—Mn2—N12^iv^	97.43 (11)	C32—O13—Mn1	90.00 (19)
O3^i^—Mn2—O12^iii^	100.34 (10)	C32—O14—Mn1	92.2 (2)
O2—Mn2—O12^iii^	152.43 (10)	C45—C41—C42	116.4 (3)
O11^iii^—Mn2—O12^iii^	56.85 (10)	C45—C41—C46	121.8 (3)
N12^iv^—Mn2—O12^iii^	93.79 (11)	C42—C41—C46	121.7 (3)
O3^i^—Mn2—N11	89.02 (11)	C43—C42—C41	119.8 (3)
O2—Mn2—N11	92.04 (11)	C43—C42—H42	120.1
O11^iii^—Mn2—N11	89.03 (12)	C41—C42—H42	120.1
N12^iv^—Mn2—N11	173.34 (11)	N1—C43—C42	124.3 (3)
O12^iii^—Mn2—N11	91.11 (12)	N1—C43—H43	117.9
O3^i^—Mn2—C31^iii^	128.57 (11)	C42—C43—H43	117.9
O2—Mn2—C31^iii^	124.34 (11)	C43—N1—C44	115.9 (3)
O11^iii^—Mn2—C31^iii^	28.56 (10)	C43—N1—Mn1	121.6 (2)
N12^iv^—Mn2—C31^iii^	96.88 (11)	C44—N1—Mn1	122.0 (2)
O12^iii^—Mn2—C31^iii^	28.30 (10)	N1—C44—C45	124.0 (3)
N11—Mn2—C31^iii^	89.57 (11)	N1—C44—H44	118.0
C2—C1—C9	119.1 (3)	C45—C44—H44	118.0
C2—C1—C11	116.8 (3)	C44—C45—C41	119.7 (3)
C9—C1—C11	124.1 (3)	C44—C45—H45	120.2
C1—C2—C3	121.6 (3)	C41—C45—H45	120.2
C1—C2—H2	119.2	C47—C46—C50	116.4 (3)
C3—C2—H2	119.2	C47—C46—C41	122.1 (3)
C4—C3—C2	121.9 (3)	C50—C46—C41	121.4 (3)
C4—C3—H3	119.1	C46—C47—C48	120.1 (3)
C2—C3—H3	119.1	C46—C47—H47	120.0
C3—C4—C10	118.9 (3)	C48—C47—H47	120.0
C3—C4—C12	117.5 (3)	N2—C48—C47	123.7 (3)
C10—C4—C12	123.6 (3)	N2—C48—H48	118.2
C6—C5—C10	121.8 (4)	C47—C48—H48	118.2
C6—C5—H5	119.1	C48—N2—C49	116.5 (3)
C10—C5—H5	119.1	C48—N2—Mn1^iv^	122.4 (2)
C5—C6—C7	120.3 (4)	C49—N2—Mn1^iv^	120.8 (2)
C5—C6—H6	119.8	N2—C49—C50	123.5 (3)
C7—C6—H6	119.8	N2—C49—H49	118.3
C8—C7—C6	120.2 (4)	C50—C49—H49	118.3
C8—C7—H7	119.9	C49—C50—C46	119.9 (3)
C6—C7—H7	119.9	C49—C50—H50	120.1
C7—C8—C9	121.7 (4)	C46—C50—H50	120.1
C7—C8—H8	119.1	C55—C51—C52	115.7 (3)
C9—C8—H8	119.1	C55—C51—C56	121.7 (4)
C8—C9—C10	118.1 (3)	C52—C51—C56	122.6 (4)
C8—C9—C1	122.7 (3)	C53—C52—C51	120.6 (4)
C10—C9—C1	119.2 (3)	C53—C52—H52	119.7
C5—C10—C9	117.9 (3)	C51—C52—H52	119.7
C5—C10—C4	122.7 (3)	N11—C53—C52	123.8 (4)
C9—C10—C4	119.4 (3)	N11—C53—H53	118.1
O2—C11—O1	124.7 (3)	C52—C53—H53	118.1
O2—C11—C1	119.1 (3)	C54—N11—C53	115.4 (3)
O1—C11—C1	116.2 (3)	C54—N11—Mn2	120.3 (3)
C11—O1—Mn1	137.1 (2)	C53—N11—Mn2	124.2 (3)
C11—O2—Mn2	144.0 (2)	N11—C54—C55	124.2 (4)
O3—C12—O4	125.1 (3)	N11—C54—H54	117.9
O3—C12—C4	117.9 (3)	C55—C54—H54	117.9
O4—C12—C4	116.9 (3)	C51—C55—C54	120.2 (4)
C12—O3—Mn2^v^	150.0 (2)	C51—C55—H55	119.9
C12—O4—Mn1^v^	131.9 (2)	C54—C55—H55	119.9
C22—C21—C29	119.6 (3)	C57—C56—C60	116.3 (3)
C22—C21—C31	120.4 (3)	C57—C56—C51	121.8 (3)
C29—C21—C31	120.0 (3)	C60—C56—C51	121.9 (3)
C21—C22—C23	121.3 (3)	C56—C57—C58	120.0 (4)
C21—C22—H22	119.4	C56—C57—H57	120.0
C23—C22—H22	119.4	C58—C57—H57	120.0
C24—C23—C22	121.0 (3)	N12—C58—C57	123.4 (4)
C24—C23—H23	119.5	N12—C58—H58	118.3
C22—C23—H23	119.5	C57—C58—H58	118.3
C23—C24—C30	119.7 (3)	C59—N12—C58	116.9 (3)
C23—C24—C32	120.6 (3)	C59—N12—Mn2^ii^	122.5 (2)
C30—C24—C32	119.7 (3)	C58—N12—Mn2^ii^	120.5 (2)
C26—C25—C30	121.4 (4)	N12—C59—C60	123.1 (4)
C26—C25—H25	119.3	N12—C59—H59	118.5
C30—C25—H25	119.3	C60—C59—H59	118.5
C25—C26—C27	120.8 (4)	C59—C60—C56	120.1 (4)
C25—C26—H26	119.6	C59—C60—H60	119.9
C27—C26—H26	119.6	C56—C60—H60	119.9

Symmetry codes: (i) *x*+1, *y*, *z*; (ii) *x*, *y*, *z*+1; (iii) −*x*+3/2, *y*−1/2, −*z*+3/2; (iv) *x*, *y*, *z*−1; (v) *x*−1, *y*, *z*; (vi) −*x*+3/2, *y*+1/2, −*z*+3/2.
